# A recommender system to quit smoking with mobile motivational messages: study protocol for a randomized controlled trial

**DOI:** 10.1186/s13063-018-3000-1

**Published:** 2018-11-09

**Authors:** Santiago Hors-Fraile, Shwetambara Malwade, Dimitris Spachos, Luis Fernandez-Luque, Chien-Tien Su, Wei-Li Jeng, Shabbir Syed-Abdul, Panagiotis Bamidis, Yu-Chuan(Jack) Li

**Affiliations:** 1 Universidad de Sevilla, Seville, Spain; 2 Maastricht University, Maastricht, The Netherlands; 3 International Center for Health Information Technology (ICHIT), Taipei Medical University, Taipei, Taiwan; 4 Aristotle University of Thessaloniki, Thessaloniki, Greece; 5 Salumedia Tecnologías SLU, Seville, Spain; 6 School of Public Health, Taipei Medical University, Taipei, Taiwan; 7 Wellcome Clinic, Taipei, Taiwan; 8 Graduate Institute of Biomedical Informatics, Taipei Medical University, Taipei, Taiwan; 9 TMU Research Center of Cancer Translational Medicine, Taipei Medical University, Taipei, Taiwan

**Keywords:** Health recommender systems, Smoking cessation, Motivational, Messages, App, Mobile, Computer-tailoring, Behavioral change

## Abstract

**Background:**

Smoking cessation is the most common preventative for an array of diseases, including lung cancer and chronic obstructive pulmonary disease. Although there are many efforts advocating for smoking cessation, smoking is still highly prevalent. For instance, in the USA in 2015, 50% of all smokers attempted to quit smoking, and only 5–7% of them succeeded – with slight deviation depending on external assistance. Previous studies show that computer-tailored messages which support smoking abstinence are effective. The combination of health recommender systems and behavioral-change theories is becoming increasingly popular in computer-tailoring. The objective of this study is to evaluate patients’s smoking cessation rates by means of two randomized controlled trials using computer-tailored motivational messages. A group of 100 patients will be recruited in medical centers in Taiwan (50 patients in the intervention group, and 50 patients in the control group), and a group of 1000 patients will be recruited on-line (500 patients in the intervention group, and 500 patients in the control group). The collected data will be made available to the public in an open-source data portal.

**Methods:**

Our study will gather data from two sources. The first source is a clinical pilot in which a group of patients from two Taiwanese medical centers will be randomly assigned to either an intervention or a control group. The intervention group will be provided with a mobile app that sends motivational messages selected by a recommender system that takes the user profile (including gender, age, motivations, and social context) and similar users’ opinions. For 6 months, the patients’ smoking activity will be followed up, and confirmed as “smoke-free” by using a test that measures expired carbon monoxide and urinary cotinine levels. The second source will be a public pilot in which Internet users wanting to quit smoking will be able to download the same mobile app as used in the clinical pilot. They will be randomly assigned to a control group that receives basic motivational messages or to an intervention group, that receives personalized messages by the recommender system. For 6 months, patients in the public pilot will be assessed periodically with self-reported questionnaires.

**Discussion:**

This study will be the first to use the I-Change behavioral-change model in combination with a health recommender system and will, therefore, provide relevant insights into computer-tailoring for smoking cessation. If our hypothesis is validated, clinical practice for smoking cessation would benefit from the use of our mobile solution.

**Trial registration:**

ClinicalTrials.gov, ID: NCT03108651. Registered on 11 April 2017.

**Electronic supplementary material:**

The online version of this article (10.1186/s13063-018-3000-1) contains supplementary material, which is available to authorized users.

## Background

Smoking is one of the leading causes of preventable deaths worldwide [1, 2]. Smoking tobacco is proven to have detrimental effects on different organs and is the root cause of several chronic diseases [3]. Among many conditions, smoking can cause cancer, heart disease, lung disease, chronic obstructive pulmonary disease, and it increases the risk of tuberculosis and other diseases related to the immune system [4, 5]. Additionally, secondhand smoking has also been linked to lung cancer, coronary heart disease, respiratory infections, middle ear disease, and sudden infant death syndrome [6].

Despite efforts to increase awareness about the harmful consequences of smoking, smoking cessation rates remain low [7–11]. Various methods have been used to promote smoking cessation in patients, including behavioral therapy, nicotine replacement therapy, and an array of medications. However, the highly addictive chemical nicotine found in tobacco usually causes intense withdrawal symptoms that make it difficult for smokers to quit [12, 13]. These symptoms include headaches, coughing, fatigue, anxiety, depression, and irritability [14]. Withdrawal symptoms act as triggers that increase the urge to smoke, which otherwise are used to relieve negative emotions such as stress [15]. Consequently, there is a high probability of relapse following smoking cessation [16, 17].

### Past studies

Among new smoking cessation methods, computer-tailored interventions have proven to be effective [18–20]. These interventions have been tested both in isolation [18, 21, 22] and in combination with existing methods [23, 24]. The relatively low cost and universal use of mobile phones make them useful to deliver these computer-tailored interventions to patients [25, 26].

With this information, the SmokeFreeBrain (SFB) project [27] assessed the success rate of different interventions for smoking cessation with respect to health economics. The SFB project studied the cost-effectiveness of these interventions and proposed a plan to implement them.

One part of theSFB project was to develop the Mobile Motivational Messages for Change intervention (from now on, 3M4Chan).

The 3M4Chan intervention measures the effectiveness of motivational messages delivered via smartphone to users who wanted to quit smoking. Utilizing the Social, Local, and Mobile (SoLoMo) study’s health-recommender system [28] as a basis, the 3M4Chan intervention uses a modified mobile application (app) to improve its usability, and an includes an inventory of messages based on the successful I-Change model of behavioral change [29–31] which better tailors the motivational messages to remain smoke free. The 3M4Chan intervention also differs from the SoLoMo study in the following aspects: duration of the intervention (6 months in 3M4Chan compared to the 12 months in SoLoMo), follow-up frequency, type of performed assessments and outcome variables, and inclusion and exclusion criteria.

### Hypothesis

We hypothesize that the patients receiving the motivational messages selected by our health recommender system (and grounded in the I-Change model) will have better smoking cessation rates than those who do not receive any messages and also better rates than those who receive motivational messages that are not based on the I-Change model.

### Need for a trial

Although previous studies have used computer-tailoring [18–20], the I-Change model [23, 32], mobile phones [25, 26], and the health recommender systems to support smoking cessation [33, 34], this is the first study that will bring together all four features. Our study will use a specially designed app called Quit and Return (有戒有還 in Mandarin; hereafter referred to as “the app”) in two methodologically different pilots – a clinical pilot and a public pilot. They will be assessed differently although they will share some common metrics.

If successful, this app could be integrated into treatments for smoking cessation, thereby promoting higher success rates in those attempting to quit. This would result in a better patient health, higher quality of life, lower incidence of smoking-related diseases, and reduced costs in the healthcare sytem.

## Methods

### Design and setting

The primary objective of this study is to assess whether tailored motivational messages supporting smoking cessation—as selected by a health recommender system and delivered by mobile phones to patients—contribute to higher smoking cessation rates.

Secondary objectives include: (1) comparing smoking cessation rates to other user metrics and technical aspects of the system; (2) investigating the relationship between usage of the app used in the 3M4Chan intervention [35] and users’ opinions of messages; and (3) determining if a relation exists between the users’ physical activity levels and their mobile app usage, and their smoking cessation rates.

Both the primary objective and the secondary objectives are common for the clinical and public pilots, although their methodologies and assessments do not completely coincide.

The present document shows the protocol version 1.0.4, 30 May 2018. Major protocol amendments, if any, will be handled by the Taipei Medical University informing any involved stakeholder, and updating the ClinicalTrials.gov registry diligently. Table 1 describes the clincal and public pilot. We followed the Standard Protocol Items: Recommendations for Intervention trials (SPIRIT) guidelines while writing this protocol manuscript (see Additional file 1).

**Table 1:** Description of the clinical and public pilot

Name of the pilot	Number of patients	Groups	Treatment	Data to be assessed
Clinical pilot (RCT; 100 patients)	50	Control	Usual care (behavioral therapy and pharmacological treatment)	Smoking cessation rate (clinically validated)
	50	Intervention	Usual care + 3M4Chan app (advanced tailored messages)	Smoking cessation rate (clinically validated)
				Recommender system
				User engagement
				Physical activity
Public pilot (RCT; 1000 patients)	500	Control	m-health (basic tailored messages)	Smoking cessation rate (self-reported)
				Recommender system
				User engagement
				Physical activity
	500	Intervention	m-health (advanced tailored messages)	Smoking cessation rate (self-reported)
				Recommender system
				User engagement
				Physical activity

*RCT* randomized controlled trial

### Target population

The target population is any smoker in Taiwan who want to quit smoking.

### Recruitment procedure

The participants in this study can be divided into two groups: those who participate in the clinical pilot and those who take part in the public pilot. Participants in the clinical pilot will be recruited from smoking cessation units at Taipei Wellcome Clinic and the Taipei Medical University Hospital in Taiwan between 1 September 2017 and 31 July 2018. They will be asked by a nurse or researcher, whether they want to be part of the study in the waiting room before their first visit to the smoking cessation unit. They will be handed written information about the reasoning of the study, and further, they have to sign an informed consent to participate (Additional file 2). The public pilot will be open to any smoker who is willing to quit. In both pilots, each participant must own an Android mobile phone, and accept the terms and conditions of the services provided by the 3M4Chan app. The participants for the public pilot will be recruited online among all the Taiwanese population between 1 September 2017 and 31 July 2018. They will be invited to join the study by downloading the app. These invitations will be done with targeted ads on Facebook and Google, as well as with retargeting banners, which have previously been proven to be successful [36]. In addition, posters and informative leaflets will be distributed at the above-mentioned smoking cessation units and at Health Promotion Administration centers in Taiwan to attract candidates in non-digital environments. A total of four posters, 200 leaflets, and 400 business card-sized advertisements will be printed to disseminate information about the app.

### Inclusion/exclusion criteria

In order to be eligible for the clinical pilot study, each patient has to meet the following inclusion criteria. Participants will be required to be a current smoker aged 20 years or older who owns an Android mobile phone and is able to read Mandarin. Additionally, only those who have smoked at least once per month for the past 2 years and are willing to share information from their medical Electronic Health Record (EHR) with project researchers will be considered. Furthermore, patients will be required to sign an informed consent and agree to be followed up for 6 months.

For the public pilot study, all interested people who download the 3M4Chan app in their Android smartphone, who are aged 20 years or older, and who can read English or Mandarin will be able to join.

### Planned interventions: control and intervention groups

In the clinical pilot, participants will be randomly allocated into two groups: Participants in the control group will receive the usual care provided at the medical center, while participants in the intervention group will be provided with a mobile app that allows them to receive tailored motivational messages in addition to the usual care. Similarly, participants in the public pilot (those who download the app but do not visit the medical center) will also be randomly allocated into two groups. Those in the control group will receive basic tailored messages without prioritization (the system sends a random message from a list of messages whose contents fit the user profile). Users in the intervention group will receive messages that are more specifically tailored to them (the message to be sent will be determined by its usefulness as reported by others with a similar user profile).

### Data management and quality assurance

Participants’ clinical data will be input by physicians into the Realsun, which is a Taiwanese EHR system [37], as is routinely done in usual care. To ensure confidentiality, the data will only be accessible by the physicians and researchers involved this project, and the Taipei Medical University Hospital will classify the data as secrets based on legal regulations.

Participant data related to mobile phone activity (including profile details participants of the public pilot, and their registered physical activity time) will be automatically stored in a secure password-protected electronic database located at the ICHIT department of the Taipei Medical University that uses PostgreSQL and is accessible only to researchers involved in this study. Additionally, participants who drop out of the study will be registered along with their reasons for doing so.

The results of this study will be published in peer-reviewed scientific journals and presented at national and international conferences. Moreover, anonymized datasets from the study will be available via a data management system and thereby accessible to third-party researchers. The system will be based on CKAN [38], an open-source data portal platform. It will be possible to use, search, share, and publish these data.

We will assure the quality of the data entry process by checking use cases during the app’s testing period: the data that are stored in the database will be evaluated to be the same that is expected values after the use case. If it were not the correct, the system would be debugged until it is fixed. Furthermore, random checks will be conducted throughout the duration of the study at a minimum frequency of once per month to ensure that the data are being stored correctly.

Only participants who meet the study’s eligibility criteria, which include signing the informed consent after a full explanation of its consequences, will be enrolled in the clinical pilot. Only participants who accept the terms and services of the app will be enrolled in the public pilot. Data will be kept for 5 years after the research is completed after which all electronic and paper copies will be destroyed.

All consultations will take place at the smoking cessation units at the Wellcome Clinic and Taipei Medical University Hospital. The data analysis will be done at the Taipei Medical University (Taiwan), and the University of Seville (Spain). The Data Monitoring Committee will be formed by researchers of these two institutions with total independence from the sponsors and with no competing interests.

No harm nor additional adverse events from regular smoking cessation care are expected as part of the clinical trials. No harm nor any adverse events are expected as part of the public trial.

The status of the trials is reported to the SmokeFreeBrain Project Consortium. The Data Monitoring Committee members are part of this consortium. It is the consortium, led by its coordinator, who may decide to terminate the trials if the monthly interim analysis show deviations from the expected plan.

### Procedure for generating texts

The motivational messages include a wide range of topics, such as physical activity recommendations, diet tips, reminders about the benefits of being a non-smoker, advice for coping with temptation, little known facts about smoking, suggestions to avoid relapses, how to develop skills required to quit effectively, smoking-related social influences, planning actions to change, quitting smoking self-efficacy expectations, and attitudes towards smoking cessation. The recommender system uses de Vries’s I-Change (or Integrated) model [29–31] to tailor messages to users and guide them toward an effective behavioral change.

The messages are designed to cover the different behavioral-change factors – determinants that the I-Change model suggests we should address in order to foster a behavior change. This process consists of two steps. The first one is checking the users’ profile, which was previously answered by the own user through a series of in-app questionnaires. These questions depict the I-Change users’ profile, providing information about their behavioral determinants (attitudes, social support, skills, self-efficacy, intentions, action planning, etc.). After that information is known, the system iterates over a set of 228 different messages that can be sent to the user. Each message is categorized in one of the 131 to different profiles types created based on the I-Change model. For instance, a message could be targeted to young female users with low social support to quit smoking, while other could be targeted to elder male users which have low self-efficacy levels to quit. Then, the system discards those messages which are incompatible with the user we are going to send the message to – for instance, messages targeted to pregnant women if the user is a male –. Finally, the system selects the message which has the highest usefulness rating by other users. The usefulness rating is recorded every time a user receives a message in their phone with a five-star scale.

### Randomization

In the clinical pilot, randomization will be done with computer-generated random numbers in the website www.randomizer.org. In the public pilot, the randomization process is handled by the server using the JavaScript random method called Math.Random().

### Methods to protect against other sources of bias

The clinical pilot component of this study is designed to be a single-blinded randomized control trial. Care providers at both medical centers will be instructed to treat patients from both groups equally and they will be responsible for patient allocation. Since they do not have any competing interest, we think it is unlikely they influence any group by the lack of blinding. Conversely, the public pilot will be double-blinded as there is no direct interaction with care providers (Table 1). In both cases, outcome assessors will be blinded although the different variables in the datasets will determine the groups.

To avoid bias in the clinical pilot, non-clinical researchers not involved in providing the intervention will be in charge of assessing the results. In the public pilot, although there are no people directly involved providing the intervention, and there is a low risk of bias in the results assessment, the analysis will be done by the same non-clinical researchers not involved in providing the clinical pilot intervention.

### Duration of treatment period and follow-up

In the both the clinical and public pilots, patients will be recruited for a period of 7 months, and treated and followed up for 6 months.

### Measures: baseline and outcome and process measures

The clinical pilot and public pilot are different interventions with shared metrics and also exclusive ones to each case. The primary outcome, the smoking cessation rates, will be measured at the 60, 120, and 180-day follow-up time points, using the urinary cotinine and expired carbon monoxide levels in the clinical pilot case, and the self-reported questionnaires in the public pilot case. To assess the secondary outcomes, different metrics are required. Table 2 includes information about how they will be calculated, to which other metrics they will be compared, and to which pilot they are related.

**Table 2:** Description of metrics to assess the primary and secondary outcome

Metric	Calculation	Comparisons	Pilot	Related secondary outcome
Primary outcome				
Smoking cessation rate	Total number of people who relapsed / total number of people in the group at 60 days, 120 days, and 180 days of their quitting date	• User engagement at an individual level • User engagement at an aggregated level • User mobile app usage • User quitting attempts • User lifestyle feedback • Physical activity	• Clinical • Public	System influence on smoking cessation and app influence on users physical activity levels
Secondary outcomes				
User engagement at an individual level	Messages read by the user / total number of messages sent to the user	• Smoking cessation rate	• Public • Clinical	System influence on smoking cessation
Engagement at an aggregated level	Mobile application rolling retention, session length distribution, session frequency, sessions per user, return rate	• Smoking cessation rate	• Public • Clinical	System influence on. smoking cessation.
User quitting attempts	Number and date of quitting attempts	• Smoking cessation rate	• Public • Clinical	System influence on smoking cessation
User app behavior	Time spent per app section	• User message ratings • User satisfaction with messages	• Public • Clinical	App usage. and opinions on messages the users received
User satisfaction with messages	Satisfaction questionnaire	• User mobile app usage • Mobile app behavior • User message ratings	• Clinical	App usage and opinions on messages the users received
User message ratings	Users’ votes for each message on a 5-star scale	• User app behavior	• Public • Clinical	App usage and opinions on messages the users received
User lifestyle feedback	Comparison of changes in user lifestyle (at baseline and after 6 months) through the questionnaires: EQ-5D-5 L, IPAQ for physical activity, and SF-36	• Smoking cessation rate • Mobile app usage	• Clinical	App influence on users physical activity levels
Physical activity	Total time (min) of activity per user, retrieved by GoogleFit	• Smoking cessation rate • Mobile app usage	• Public • Clinical	App influence on users physical activity levels

*EQ-5D-5 L* EuroQol 5-dimension 5-level questionnaire, *IPAQ* International Physical Activity Questionnaire, *SF-36* Short Form 36-item Health Survey

Although they are not necessary to the assessment of the primary and secondary outcomes, the metrics described in Table 3 will also be retrieved and analyzed because they are considered relevant and potentially useful.

**Table 3:** Description of additional metrics to be measured in the study

Metric	Calculation	Pilot
User reliability	Comparison of the abstinence self-report at 2-week intervals with the measurements of the CO-oximeter	Clinical
QALY (financial aspects)	Healthcare resource utilization and cost analysis (cost of devices used, pharmacological treatment and time spent for various purposes)	Clinical
Precision of the recommender system	Messages sent and rated more than four stars / total number of rated messages	Public Clinical
Smoke-free period	Time range between quitting date and the last smoke-free report	Clinical

*CO* carbon monoxide, *QALY* quality-adjusted life year

### Statistical analysis

In the clinical pilot, patients will be classified as either smokers or non-smokers according to their positive or negative values in the urine test and the expired CO tests (PiCO values between 0 and 6 will be considered non-smokers) at each follow-up visit. In case of discrepancy between tests, we will consider the patient as a smoker. We will perform repeated analysis of variance (ANOVA), and provide *p* values, to compare the number of non-smokers in the intervention versus control group at 2 months, 4 months and 6 months of their quitting day.

In the public pilot, users will be considered to be non-smokers according to their self-reported answer to the question: “Are you still resisting the temptation or have you smoked? Please, be honest.” We will perform repeated ANOVA, and provide *p* values, to compare the number of non-smokers in the intervention versus control group every 2 weeks.

For secondary outcomes, the numerical variables (user engagement at aggregated level, user mobile app usage, user quitting attempt, user app behavior, and user lifestyle feedback EuroQoL 5 dimensions 5 levels questionnaire (EQ-5D-5 L) and Short Form 36-item Health Survey (SF-36), and physical activity) will be assessed with *T* tests and variance ANOVA tests. Categorical variables (user engagement at individual level, user satisfaction with messages, user message ratings, and lifestyle answers to the International Physical Activity Questionnaire (IPAQ)) will be assessed with chi-square tests. In addition, we intend to study the combination of all metrics with a linear regression analysis, and the Kaplan-Meier method to analyze the overall survival rate (non-smokers). All tests will be two-tailed and with a significance level set at 0.05. All analyses will be done according the intention-to-treat principle, and using the SPSS software version 25. Missing data will be calculated with the last observation carried forward.

### Sample size and power calculations

Calculations regarding the needed number of participants to measure the primary outcome – smoking cessation rates – have been done to ensure the statistical significance of the results.

For the clinical pilot, accepting an alpha risk of 0.05 and a beta risk of 0.2 in a two-sided test, 45 subjects will be necessary in both the first group and second groups in order to find a statistically significant proportion difference, which is expected to be 0.2 and 0.5 in the first and second groups, respectively.

For the public pilot, accepting an alpha risk of 0.05 and a beta risk of 0.2 in a two-sided test, 489 subjects are necessary in first group and 489 in the second to find as statistically significant a proportion difference, expected to be of 0.07 in group 1 and 0.14 in group 2.

A dropout rate of 15 and 20% is anticipated for the clinical and public pilot, respectively. Although other studies using computer-tailored interventions have reported higher dropout rates, this study will consider participants who do not return for the consultation at month 6 to be failure cases. Consequently, the anticipated dropout rates include only those who withdraw for reasons other than relapse.

### Planned recruitment rate

**Fig. 1:**
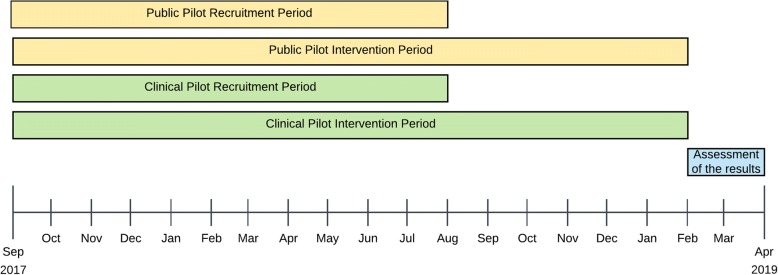
Difference of enrollment, intervention, and assessments periods in the clinical and public pilots

**Fig. 2:**
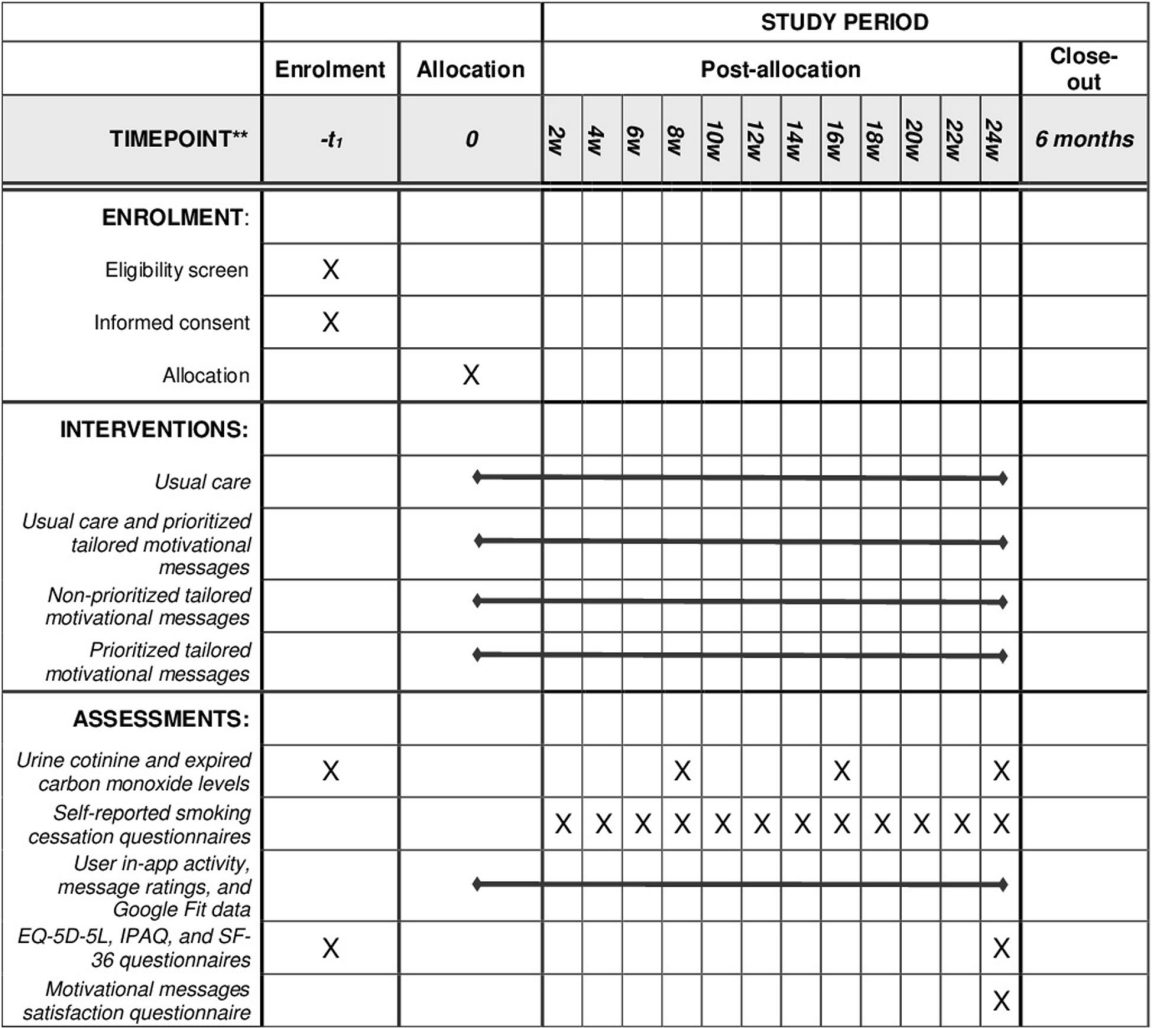
Standard Protocol Items: Recommendations for Interventional Trials (SPIRIT) Schedule of enrollment, interventions, and assessments

The clinical pilot recruitment rate is expected to be 17 per month in average, with the exception of February 2018 in which we do not expect to have recruit patients due the Chinese new year festivities. In the public pilot, we the planned recruitment rate is 700 users in the first 4 months, and 300 users in the three remaining months. This difference in the rate is due to the fact that we plan to conduct a digital marketing campaign to promote the app in the first 4 months, and that we expect a reduced number of new users in February coinciding with the Chinese new year. Figure 1 summarizes the difference in the recruitment, execution, and assessment periods for both pilots, and Fig. 2 details it.

### Materials

The clinical pilot will make use of all the materials normally used to treat patients at the smoking cessation units at Taipei Welcome Clinic and Taipei Medical University Hospital.

Carbon monoxide levels will be tested using a PiCO Smokerlyzer CO-oximeter; a value range of 0–6 will be considered normal for a non-smoker. Similarly, a Safecare Biotech COT Rapid Test device, which contains line indicators for positive and negative test results, will be used to measure urinary cotinine levels.

Several smoking cessation medications will be used during the trial: Nicotinell TTS 20 and TTS 30 patches, Nicotinell 2-mg chewing gum and 10-mg inhalers, Buporin 150-mg sustained-release tablets, and Champix 0.5-mg and 1-mg tablets. The dosage and duration of each medication will be prescribed by the physician, and the patients will be charged NT$200 per consultation.

**Fig. 3:**
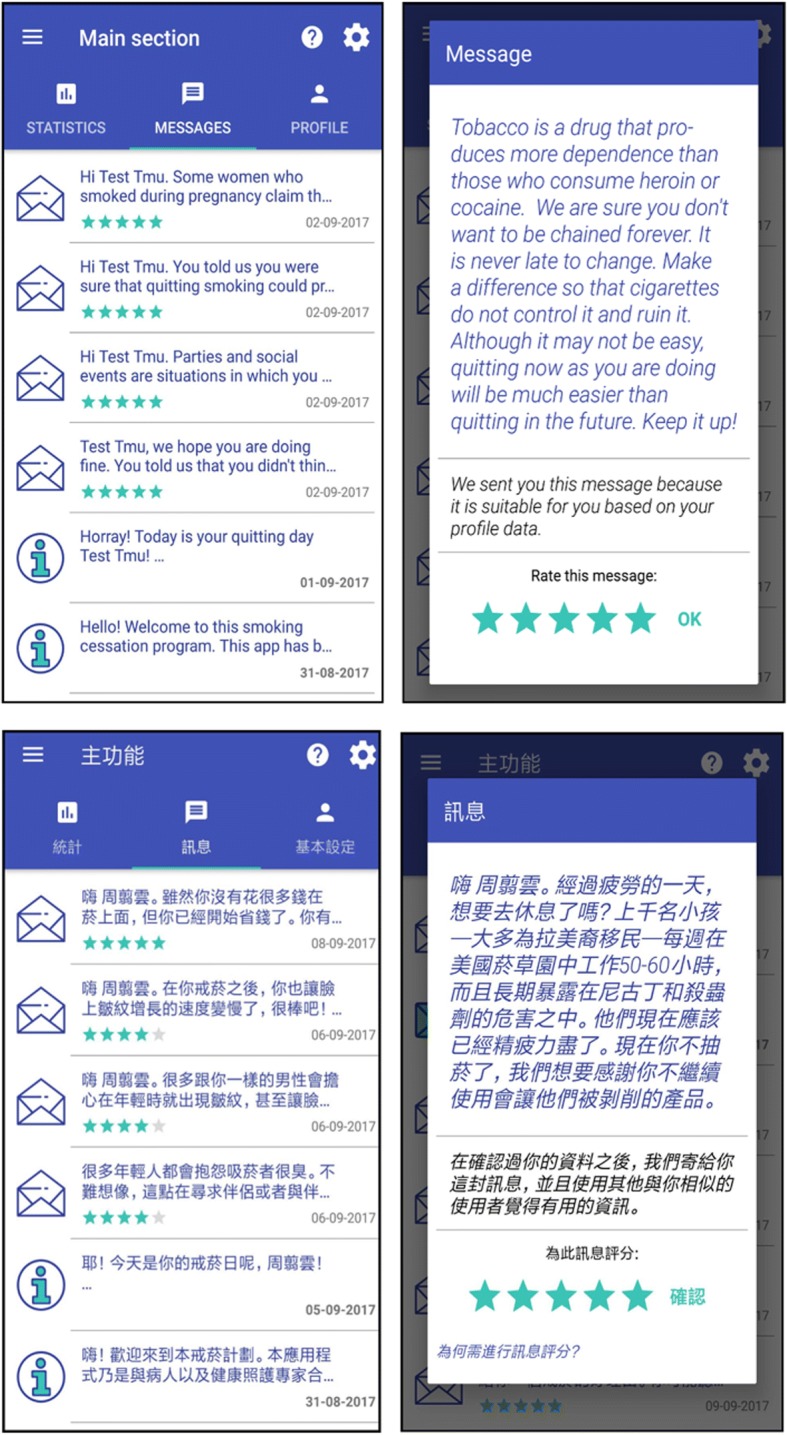
Screenshots of the app (English and Mandarin versions) showing different sections

In addition, our specially designed mobile app will be used to deliver motivational health messages. Figure 3 shows its interface in both English and Mandarin. The app has been programmed in Android’s native language. It uses Mirth Connect as its communication channel [39], Firebase for platform notifications, and PostgreSQL as its database management system.

A nurse will provide patients with instructions for downloading and using the app. Questions related to the patient’s basic demographic information and an extended profile (including social influences, plan of action, skills, attitudes, and self-efficacy with regard to smoking) will be assessed in the app.

The data obtained from the database will be analyzed in a comma-separated value (CSV) format using SPSS software.

Users’ physical activity data will be collected using their mobile phones. The mobile app will use GoogleFit [40] to track daily physical activity. These data will be available to users through the GoogleFit app but not displayed within the 3M4Chan app, as it will be utilized for research purposes only.

Patients will be followed up after 60, 120, and 180 days of their initial consultation with a variance margin of 5 days. In the initial consultation, the patient will be assessed in the smoking cessation unit after they have been referred from either the pneumology unit or another clinical department. Smoking-related symptoms, treatment adherence, daily cigarette use, and possible adverse events will be discussed and exhaled carbon monoxide, urinary cotinine, and other clinical information, such as weight and height, will be assessed. Quality of life (EQ-5D-5 L, IPAQ, and SF-36) questionnaires will also be collected. The patient will be instructed on relaxation techniques and on tips to avoid smoking.

During the second consultation, physicians will gather patient information on smoking-related symptoms, treatment adherence, daily cigarette use, possible adverse events, exhaled carbon monoxide and urinary cotinine levels, and other clinical information. Coaching for relapse prevention will be performed.

At the third consultation, information about smoking-related symptoms, treatment adherence, possible adverse events, exhaled carbon monoxide and urinary cotinine levels, and other clinical information will again be collected. New relaxation techniques will be explained if the previously taught techniques have not been effective. Techniques for relapse prevention will be reviewed. It will also be collected the users’ physical and psychological health state, their exhaled carbon monoxide levels, the results from a urinary cotinine test, and will be asked clinical information such as weight and height.

At the fourth consultation, physicians will collect information about smoking-related symptoms, treatment adherence, and possible adverse events. Techniques for avoiding the risk of relapse are reinforced. Data about blood pressure, weight, height, and quality of life (using EQ-5D-5 L, IPAQ, and SF-36 questionnaires as well as a questionnaire assessing the satisfaction about the motivational messages received through the 3M4Chan app) will also be collected. All input clinical data will be checked for correctness by two healthcare professionals to ensure quality.

The public pilot will use the same mobile app as the clinical pilot. No additional materials will be used.

## Discussion

### Expected outputs for validation of our hypothesis

Recently, several recommender systems have been implemented to support smoking cessation [33, 41, 42]. We expect that our recommender-system-aided motivational messages will help increase user engagement at both the individual and aggregate level and lead to prolonged periods of smoking abstinence in accordance with our hypothesis. This will be validated by the metrics measured over the course of our study, such as the health recommender system precision, smoking abstinence, app usage statistics, and user feedback.

### Expected outcomes

We hope to provide a new method to achieve smoking cessation that will be integrated into the usual care received by patients. This can either come from just using the mobile app in the public intervention, or by combining the mobile app with the pharmacological treatment in the clinical intervention setting. Despite being different approaches, achieving an increase to smoking cessation rates will help to reduce the incidence of diseases directly related to smoking as well as those arising from exposure to secondhand smoke. This will lead to increased life expectancy and improved life quality.

### Dissemination of the results

The results of this study will be published, and a subset of the retrieved data will be made public as part of the SmokeFreeBrain project’s database. Public datasets will be fully anonymized and protected in accordance with directives 2002/58/EC [43] and 95/46/EC [44], which regulate the protection of privacy in electronic communications and the processing and free movement of personal data.

The data will be made available through an open-source data management portal based on CKAN. Third-party researchers and organizations will have access to both the raw data (where anonymization has made this possible) and to a rich set of metadata for each dataset. In this portal, users will be able to search up to field level different keywords and tags to browse related datasets, and see their format, availability, and licensing type. The published datasets will be a valuable resource for future studies, and we intend this portal become a base reference point for future related studies.

### Problems anticipated

The dropout rate among participants is one of the main challenges we anticipate for this study. Patients might dropout by ceasing to share EHR data, by terminating their use of the app, or by becoming unresponsive to follow-ups over the course of the trial.

### Ethics

This study has been approved by the Ethical Committee of Taipei Medical University-Joint Institutional Review Board (TMU-JIRB) and has been registered at ClinicalTrials.gov with ID: NCT03108651. The study includes all items from the World Health Organization Trial Registration Dataset (Additional file 3).

### Informed consent

Patients in the clinical pilot will sign an informed consent. All patients using the app, regardless of which pilot they are participating in, will be asked to read and accept the terms and conditions (http://13.114.86.199/policy-en.html), which explain the usage of their data and app analytics, the rights they have during the pilot, and the offered services by the pilot 3M4Chan app in detail.

### Budget

The SmokeFreeBrain project has received funding from the European Union’s Horizon 2020 research and innovation programme under grant agreement No. 681120 with a total amount of €2,981,001. The Taipei Medical University, is funded by the Ministry of Science and Technology, Taiwan, under the Grant number 106–2923-E-038-001-MY2, with a total amount of €206,863.95. You can contact the SmokeFreeBrain project sponsor CORDIS at + 32,229–91,111, and the MOST form Taipei Medical University at + 886–2–2737-7992.

### Trial status

Currently recruiting.

## Additional files

Additional file 1: Standard Protocol Items: Recommendations for Interventional Trials (SPIRIT) 2013 Checklist: recommended items to address in a clinical trial protocol and related documents*. (DOC 120 kb)

Additional file 2: Informed consent. (DOCX 39 kb)

Additional file 3: World Health Organization (WHO) Dataset. (DOCX 31 kb)

## Abbreviations

3M4Chan: Mobile Motivational Messages for Change
App: Mobile application
EHR: Electronic Health Record
QALYs: Quality-adjusted life years
RCT: Randomized controlled trial
SFB: SmokeFreeBrain
SoLoMo: Social, Local, and Mobile
