# Supplementary material for: A recommender system to quit smoking with mobile motivational messages: study protocol for a randomized controlled trial
Source: Trials. 2018 Nov 9;19:618. doi: 10.1186/s13063-018-3000-1 (PMC6230227; doi:10.1186/s13063-018-3000-1)
Supplement: Supplementary file 2 — Informed consent. (DOCX 39 kb) [file 13063_2018_3000_MOESM2_ESM.docx]

Informed Consent to be signed by the patients:

**TMU-Joint Institutional Review Board**

| **Name of the Trial：**Mobile Motivational Messages for Change (3M4Chan) intervention in TMU |
| --- |
| **Implementation Institution:** Taipei Medical University Hospital, Graduate Institute of Biomedical Informatics, Address:  **Principal investigator:** Dr. Syed Abdul Shabbir, Assistant Professor & Health IT Consultant, TMU  **Co-PI:** Professor Yu-Chuan (Jack) Li, Professor and Dean, COMST, TMU, (02)6638-2736#1513  Dr. Chien-Tien Su, Associate Professor, School of Public Health, TMU and Director of Family Medicine, TMU Hospital, (02)2736- 1661#6525/ (02)2738-4831  Dr. Hao-Yi Fang, Doctor of Family Medicine, TMU Hospital and Director of Occupational Medicine, TMU Hospital, 0970405266  Dr. Wei-Li Jeng, Wellcome Clinic, +886 -2-2309-2807  **Contact person in case of emergency:** Dr. Syed Shabbir Abdul, Assistant Professor and Health IT Consultant, Graduate Institute of Biomedical Informatics, Taipei Medical University, Office phone number: +886 (02)6638-2736 Ext 1514, Mobile: +886989478600 |
| **Name of participant： Gender：**  **Date of Birth： Age：**  **Chart Number：**  **Mail Address：**  **Phone Number：** |
| **Name of contact person in case of emergency/relationship to the participant：**  **Mail Address：**  **Phone Number：** |
| **1. Background for the trial/research**  About 100 (50 from Taipei Medical University Hospital and 50 from Wellcome Clinic) current smokers will be recruited who contact smoke cession clinics in teaching hospitals of Taipei Medical University (TMU) and Wellcome Clinic. They will be randomly assigned either to Usual Care (UC) or Usual Care plus 3M4Chan Intervention group (IG). A randomized comparative effectiveness trial will be conducted. T8.5.1. Patient will enroll from Sept 2017 to March 2018. In UC group, the physician provides a brief advice to quit during first consultation, a quit plan template, and medication prescription (Varenicline, Bupropion, Champix, Nicotinell TTS20) as decided by the physician. IG group includes UC plus a mobile App that is programmed to push tailored messages for health concern and readiness to quit, tips for sustaining abstinence, use of interactive self-assessments, helpful cessation information[S1]. This group of smokers will benefit from tailored cessation interventions with integrated body weight management elements that take into consideration the prevailing local and cultural influences on diet and levels of physical activity. IG will be continued even after stopping of the UC. |
| **2. Purpose for the trial/research** The purpose of this study will be to explore methods effecting successful abstinence in the candidates willing to quit smoking when compared with usual care. |
| **3. Inclusion and exclusion criteria Inclusion criteria:**  · Age 20 years or older and attend to smoke cessation clinic at TMU hospital or Wellcome clinic  · Have an Android mobile phone  · Able to read Traditional Chinese  · Willing to participate in every phases of the research  · Willing to be followed-up for 6 months  · Smoked at least once every month in the last two years  · Current smoker  **Exclusion criteria:**  · Under 20 years old.  · Not willing to participate in the trial  · Those who do not have Android mobile phone or unable to read Traditional Chinese  · Not willing to share medical information from EHR  · Subjects who developed adverse effects related to the pharmacological treatment included in the trial |
| **4. Procedures and relevant tests for the trial/ research:**  This intervention will use mobile messaging to support subjects quit smoking in addition to normal therapy. We will use urine cotinine and CO test. For urine cotinine test, we will use a strip to test the urine sample collected in the test tube (very minimal and non-invasive) and CO will be measured by breathing. We will require the patients to complete the following questionnaires:  EQ-5D-5L, SF-36, IPAQ27 questionnaire: At first consultation and 6 months  Satisfaction questionnaire: At 6 months  *If the trial/research needs to conduct subjects’ HIV testing, the agreement from the related party must be obtained according to the law. Please also add the following explanation.*  Due to the requirement of the test in excluding those who are infected HIV, a HIV testing will be conducted in the named by confidential approach. If the testing result is negative, the subject could join the test; however, if it is positive (including false positive), the test will provide follow-up medical treatment referral or counselling. Once it is confirmed, it must be reported to the management authority according to law (HIV Infection Control and Patient Rights Protection Act).  I agree to receive the above test and the relevant medical referral, counselling, and reporting to the management authority when necessary.  Signature: ______________ Date: _________ (year/ month/ day) |
| **5. Possible adverse effects and damage, and standard procedures for handling**  No adverse effect will occur. Standard procedure and safety will ensure for using urine cotinine and CO test. Urine cotinine test will use a strip to test urine sample collected in the test tube. It’s non-invasive, and CO test will be conducted by breathing of the respondent. |
| **6. Expected effects/results from the trial/research**  Effectiveness of mobile apps to quit smoking |
| **7. Alternative treatment options** N/A |
| **8. Things should be avoided or restricted during participating in the trial/research**  Participants should be protected from avoidable harm. |
| **9.Confidentiality**  Taipei Medical University Hospital **will classify data as secrets based on legal regulations. You understand that Ministry of Health and TMU-JIRB have the right to review your data under ethical requirement for confidentiality.**  **All test results and diagnoses made in this trial/research will be labeled with a project serial number and your name will be taken off from all labels. We will keep your privacy, while the above-mentioned institutes have the right to review your data according to legal regulations. Your identification will be confidential in all publications associated with this trial/research.**  *If the research is the case which is supervised by USA FDA, USA FDA might check the relevant records and also put the relevant testing information on the website of Clinicaltrial.gov (*[*http://www.ClinicalTrials.gov*](http://www.clinicaltrials.gov)*). The content of Clinicaltrial.gov website won’t include any identifiable information, and the abstract of research outcome will be provided on the website. You can search the information on the website at any time. The research executed in EU or European Economic Area (EEA) also might be requested to put the relevant testing information on the website of Clinicaltrialsregister.eu (*[*http://www.clinicaltrialsregister.eu*](http://www.clinicaltrialsregister.eu)*). You can search the information on the website anytime.* |
| **10. Withdrawal/termination of the trial/research, and disposal of specimens and related information in such conditions.**  **Description:** The principal investigator should list legal ways for specimens and information disposal if a participant withdraws from the trial/research, and ask for participant’s decision and permission.  *The decision of participation in the current trial/research is yours, and you may withdraw your permission at any time during the trial/research without any reason. There will be no negative impacts on your continual health care by the current physician and hospital. In addition, by signing this consent form you also understand the possibility that principal investigator or the sponsor may halt the trial when necessary. This is not going to post any negative impacts on your continual care by the current physician and hospital either.*  *Your specimen will be executed according to your choice, but the information related to you that was obtained before you dropped out will still be kept and analyzed. Taipei Medical University Hospital or principal investigator (Dr. Syed Abdul Shabbir) will have the right to use and development. If it exceeds original range of use, you must be informed again for your agreement; meanwhile, the exceeded original range of use must be reviewed and approved in advance by Taipei Medical University- Joint Institutional Review Board.*    *Specimens：*  *🗹 I agree to provide the specimens for continual use in this trial/research. Please follow the original protocol for disposal. If it exceeds original range of use, you must be informed again for your agreement; meanwhile, the exceeded original range of use must be reviewed and approved in advance by Taipei Medical University- Joint Institutional Review Board. Please dispose or destroy it according to the fixed schedule by the end of the research.*  *🗹 I agree for the principal investigator to destroy the specimens that I provided after withdrawal (at my own decision or as recommended by the principal investigator).*    *The decision of participation in the current trial/research is yours, and you may withdraw your permission at any time during the trial/research without any reason. There will be no negative impacts on your continual health care by the current physician and hospital. In addition, by signing this consent form you also understand the possibility that principal investigator or the sponsor may halt the trial when necessary. This is not going to post any negative impacts on your continual care by the current physician and hospital either.*  *If genetic tests of your specimens are completed at the time of your withdrawal from this trial/research, the information will be kept and analyzed as described in this informed consent. XX (name of the sponsor), OOO (name of the hospital) and OOO (name of the principal investigator) will have all rights on the use and copyright of these results. Your specimens will be destroyed upon your withdrawal.* |
| **11. Damage compensation and insurance: N/A (This study will use mobile SMS for quit smoking and collect urine in test tube(non-invasive) and CO test by breathing. Therefore, there won’t be any health hazard for individual.)**  **(1)** **Sponsor of the current project (Taipei Medical University Hospital) is liable to compensation for damage directly related to the use of agent, medical device or new therapeutic techniques in this trial/research as described in the previous sections. Damage includes all adverse events, side effects or injury. Please inform principal investigator if any adverse event, side effect or injury that is directly related to the current trial/research happens. Taipei Medical University Hospital will provide medical care. You are exempt from medical care expenses for adverse event, side effect or injury that is directly related to the current trial/research.**  **(2)** **You will not lose any legal rights by signing this informed consent form.**  **(3)** **This project is (or is not) covered by insurance. If you do not want to expose to the risk, you may deny the enrollment or withdraw your agreement anytime during the trial period. For such decisions we will not ask for a reason and your rights will not be affected.** |
| **12.Rights and obligations of the participant.**  **(1)** **All cost related to the trial/research is covered by the current trial/research.**  **(2)** **During the trial/research, we will timely provide all significant findings that are related to your personal well-being and that may change your willingness to stay in the trial/research.**  **(3)** **During the trial/research period, you will be referred to Dr. Chien-Tien Su or Dr. Hao-Yi Fang in Taipei Medical University Hospital or Dr. Wei-Li Jeng in Wellcome Clinic for research-related medical care. Please feel free to contact Dr. Syed Abdul Shabbir, Taipei Medical University at Contact No. +886 2-6638-2736 Ext 1514, Mobile: +886989478600. if there is any question during the research period.**  **(4)** **If you have any question on the nature of this research, on participants’ rights, or on the possible damage directly related to participation in the trial/research, please contact TMU-JIRB at any time at 02-27361661 ext 8898 or email: tmujirb@gmail.com.**  **(5)** **Besides the above situation, any individual who is unaffiliated with the research study such as current, prospective, or past research participants or their designated representatives, permits to discuss problems and concerns; obtain information from TMU-JIRB, we can also offer input with an informed individual who is unaffiliated with the specific research protocol or plan. If necessary, please contact TMU-JIRB at 02-27361661 ext. 8898 or email: tmujirb@gmail.com.** |
| **13.Signature**  **Disclaimer from participant**  **I have been explained about the above information, and have been provided opportunities to clarify all questions related to the trial/research. I understand and agree to participate in the trial/research, and a copy of the informed consent has been provided to me. I will contact Dr. Syed Abdul Shabbir at Taipei Medical University if there is any question in the future.**  Name of participant (print)________________________  Date of birth ________________________  Signature ________________________  Date ________________________    Name of the proxy when applicable (print)__________________ (If applicable)  Signature ________________________  Relationship to participant  Date ________________________    For any person who is asked to sign this informed consent because participant himself/herself or his/her legal proxy can not sign this informed consent at this time, please print your name and specify your relationship to the participant.  Name (Print)________________________  Relationship to the participant_____________________ **(Please see regulations for the current trial/research on who is eligible to sign the informed consent if he/she is not the participant himself/herself nor the legal proxy.)**  National ID Number________________________  Contact number________________________  Mail Address_______________________________________________  Signature________________________  Date________________________    **Disclaimer from the Principal investigator**  **I guarantee that either I or an authorized member in my research team has explained about the purpose, procedure, possible risks and benefits to the person listed above. All questions have been properly answered.**  Name of the PI (Print)Dr. Syed Abdul Shabbir  Signature_____________________  Date_____________________  Name of the researcher that explains about the research (print)______________  Signature_____________________  Date _____________________  **Witness for orally informed consent**  **(If the participant cannot read the above documents, and the full document is explained by a researcher orally, a witness is required for the process.)**  **I hereby certify the principal investigator (or a researcher in the trial/research) has thoroughly explained about the research to the participant.**  Name of the witness (Print)_______________________ **(Persons related to this research cannot be the witness)**  National ID number________________________  Contact number________________________  Mail address_____________________________________________  Signature________________________  Date________________________  ◎If subjects, legal representatives, auxiliary personnel or anyone who has the right to agree all have difficulty to read, the discussion related to the agreement of the subject should be fully participated in by a witness. After confirming the agreement of subjects, legal representatives, auxiliary personnel or anyone who has the right to agree are all out of their free will, a signature should be put on the agreement as well as remark the date. Testing/ research relevant personnel must not be the witness. |
