# Supplementary material for: A recommender system to quit smoking with mobile motivational messages: study protocol for a randomized controlled trial
Source: Trials. 2018 Nov 9;19:618. doi: 10.1186/s13063-018-3000-1 (PMC6230227; doi:10.1186/s13063-018-3000-1)
Supplement: Supplementary file 3 — World Health Organization (WHO) Dataset. (DOCX 31 kb) [file 13063_2018_3000_MOESM3_ESM.docx]

# **WHO Data Set**

**Primary Registry and Trial Identifying Number**

Name of Primary Registry: TMU-Joint Institutional Review Board

Unique ID number assigned by the Primary Registry to this trial: N201702069.

**Date of Registration in Primary Registry**

Date when trial was officially registered in the Primary Registry.: 18 March 2017

**Secondary Identifying Numbers**

Other identifiers besides the Trial Identifying Number allocated by the Primary Registry, if any. These include:

The Universal Trial Number (UTN) (N/A)

Identifiers assigned by the sponsor (record Sponsor name and Sponsor-issued trial number (e.g. protocol number)) (N/A)

Other trial registration numbers issued by other Registries (both Primary and Partner Registries in the WHO Registry Network, and other registries) (N/A)

Identifiers issued by funding bodies, collaborative research groups, regulatory authorities, ethics committees / institutional review boards, etc.: ClinicalTrials, NCT03108651

**Source(s) of Monetary or Material Support**

European Union's Horizon 2020 research and innovation programme under grant agreement No 681120.

The Taipei Medical University, is funded by the Ministry of Science and Technology, Taiwan, under the Grant number 106-2923-E-038-001-MY2.

1. **Primary Sponsor**
2. Ministry of Science and Technology, Taiwan
3. **Secondary Sponsor(s)**

European Union's Horizon 2020 research and innovation programme

1. **Contact for Public Queries**

Shwetambara Kekade

Email; sv14.kekade@gmail.com

International Center for Health Information Technology

Taipei Medical University

15F., No. 172-1, Sec. 2, Keelung Rd.,

Da'an Dist., Taipei, Taiwan

Tel. Office: +886 2-6638-2736  Extn.1521

**Contact for Scientific Queries**

There must be clearly assigned responsibility for scientific leadership to a named Principal Investigator. The PI may delegate responsibility for dealing with scientific enquiries to a scientific contact for the trial. This scientific contact will be listed in addition to the PI.

Dr. Shabbir Syed Abdul

Email: drshabbir@tmu.edu.tw

Graduate Institute of Biomedical Informatics

Taipei Medical University

15F., No. 172-1, Sec. 2, Keelung Rd.,

Da'an Dist., Taipei, Taiwan

Tel. Office: +886 2-6638-2736  Extn.1514

- - Email address, telephone number, postal address and affiliation of the contact for scientific queries about the trial (if applicable). The details for the scientific contact may be generic (that is, there does not need to be a named individual): e.g. a generic email address for research team members qualified to answer scientific queries.

1. **Public Title**.

Mobile Motivational Messages for Change (3M4Chan) Intervention in TMU

1. **Scientific Title**

Mobile Motivational Messages for Change (3M4Chan) Intervention in TMU

1. **Countries of Recruitment**

Taiwan

1. **Health Condition(s) or Problem(s) Studied**

Smoking cessation

1. **Intervention(s)**
   For each arm of the trial record a brief intervention name plus an intervention description.

- Active Comparator: Case
- Motivational message will be administrated.
- Other: Case
- Motivational message will be administrated.
- No Intervention: Control
- Motivational message will not be administrated.

Intervention Name:

Mobile Motivational Messages for Change (3M4Chan) Intervention in TMU

Intervention Description:

The participants in this study will be divided into two groups: those who will participate in the clinical pilot and those who will take part in the public pilot. Participants in the clinical pilot will be recruited from smoking cessation units at Taipei Wellcome Clinic and the Taipei Medical University Hospital in Taiwan between September 1^st^ 2017 and July 31^st^ 2018. In order to be eligible for the study, each patient has to meet the following inclusion criteria. Participants will be required to be a current smoker aged 20 years or older who owns an Android mobile phone and is able to read Mandarin. Additionally, only those who have smoked at least once per month for the past 2 years and are willing to share information from their medical Electronic Health Record (EHR) with project researchers will be considered. Furthermore, patients will be required to sign an informed consent and agree to be followed up with for 6 months. Conversely, the public pilot will be open to any smoker who is willing to quit, owns an Android mobile phone, and accepts the terms and conditions of the services provided by the 3M4Chan app.

The participants for the public pilot will be recruited online among all the Taiwanese population between September 1^st^, 2017 and March 30^th^, 2018. They will be invited to join the study by downloading the app. All interested people will be able to join. There are no exclusion criteria. These invitations will be done with targeted ads on Facebook on Facebook and Google, as well as with retargeting banners, which has previously been proven to be successful [35]. In addition, posters and informative leaflets will be distributed at the above-mentioned smoking cessation units and at Health Promotion Administration centers in Taiwan to attract candidates in non-digital environments. A total of four posters, two hundred leaflets, and four hundred business card-sized advertisements will be printed to disseminate information about the app.

If the intervention is one or more drugs then use the International Non-Proprietary Name for each drug if possible (not brand/trade names). For an unregistered drug, the generic name, chemical name, or company serial number is acceptable. (N/A)

If the intervention consists of several separate treatments, list them all in one line separated by commas (e.g. "low-fat diet, exercise").(N/A)

For controlled trials, the identity of the control arm should be clear. The control intervention(s) is/are the interventions against which the study intervention is evaluated (e.g. placebo, no treatment, active control). If an active control is used, be sure to enter in the name(s) of that intervention, or enter "placebo" or "no treatment" as applicable. For each intervention, describe other intervention details as applicable (dose, duration, mode of administration, etc). (N/A)

**Key Inclusion and Exclusion Criteria**

**Clinical pilot inclusion criteria**

Age 20 years or older and attend to smoke cessation clinic at TMU hospital or Wellcome clinic

· Have an Android mobile phone

· Able to read Traditional Chinese

· Willing to participate in every phases of the research

· Willing to be followed-up for 6 months

· Smoked at least once every month in the last two years

· Current smoker

**Clinical pilot exclusion criteria**

· Under 20 years old.

· Not willing to participate in the trial

· Those who do not have Android mobile phone or unable to read Traditional Chinese

· Not willing to share medical information from EHR

· Subjects who developed adverse effects related to the pharmacological treatment included in the trial

1. **Study Type**
   Study type consists of:
   - Type of study (interventional or observational): Interventional
     - Method of allocation (randomized/non-randomized): Randomized
     - Masking (is masking used and, if so, who is masked): Masking: single ( for participants, researchers do not disclose about mobile app and messages to the control group and viceversa)
     - Assignment (single arm, parallel, crossover or factorial): Parallel Assignment
     - Purpose: Explore Successful Method to support smoking cessation.

In the clinical pilot, sequence generation will be done using a software for these purposes (www.randomizer.org). Allocation concealment is done using the previously mentioned software, as there is no way for the researcher to know what is the next result coming from the software. In the public pilot, an automatized random seed generated in a web server determines the patient randomization. No researchers are involved in this allocation result.

1. **Date of First Enrollment**
2. Anticipated or actual date of enrollment of the first participant: 3rd Oct 2017
3. **Target Sample Size**
4. Number of participants that this trial plans to enrol in total: 100
5. **Recruitment Status**
6. Recruitment status of this trial: Recruiting
7. **Primary Outcome(s)**

Outcome name: Smoking cessation rate at 2 months

Metric / method of measurement: Smoking cessation rate will be measured as the ratio of the total number of patients who relapsed with total number of patients in their group. Patients will be considered to have relapsed if they fail at least one of their urine cotinine and carbon monoxide tests.

Timepoint: 2nd month after enrollment of each respondent

Outcome name: Smoking cessation rate at 4 months

Metric / method of measurement: Smoking cessation rate will be measured as the ratio of the total number of patients who relapsed with total number of patients in their group. Patients will be considered to have relapsed if they fail at least one of their urine cotinine and carbon monoxide tests.

Timepoint: 4th month after enrollment of each respondent

Outcome name: Smoking cessation rate at 6 months

Metric / method of measurement: Smoking cessation rate will be measured as the ratio of the total number of patients who relapsed with total number of patients in their group. Patients will be considered to have relapsed if they fail at least one of their urine cotinine and carbon monoxide tests.

Timepoint: 6th month after enrollment of each respondent

1. **Key Secondary Outcomes**
   - Name of outcome: User engagement at an individual level
   - Method of measurement: Engagement at individual level will be assessed based on the rate of read messages by the patients. This is calculated as the quotient between the messages the patients have read, and the total number of messages the system has sent to the patients. This metric will be assessed throughout the observation until its end.
   - The timepoint(s) of interest: End of 6 months from enrollment
   - Name of outcome: Engagement at aggregated level - Mobile application rolling retention

Method of measurement: The percentage of users still active N days after installation. This is a ratio of the number of users whose last day of activity is past day N to the number of users who could have been active on day N. This metric will be assessed throughout the observation until its end.

- - The timepoint(s) of interest: End of 6 months from enrollment
  - Name of outcome: Engagement at aggregated level - Mobile application session length distribution
  - Method of measurement: The session length is defined as the length of time between the start of the application event and the end of the application event. The session length determines the engagement as it is relevant to know how much time patients spend in the app per session. This metric will be assessed throughout the observation until its end.
  - The timepoint(s) of interest: End of 6 months from enrollment
  - Name of outcome: Engagement at aggregated level - Mobile application session frequency
  - Method of measurement: The session frequency is a measure of how often each unique patient used the app within a given time interval. This metric will be assessed throughout the observation until its end.
  - The timepoint(s) of interest: End of 6 months from enrollment
  - Name of outcome: Engagement at aggregated level - Mobile application sessions per user
  - Method of measurement: A session is one use of the mobile application by a patient. This begins when the application is launched and ends when the application is terminated. This metric will be assessed throughout the observation until its end.
  - The timepoint(s) of interest: End of 6 months from enrollment
  - Name of outcome: Engagement at aggregated level - Mobile application return rate
  - Method of measurement: Return rate measures the percentage of patients who return to the app on a specific time after installation. It is measured by cohort group - that is, based on when patients first opened the app. It is calculated as the ratio of the number of users active on a given period to the size of the cohort. This metric will be assessed throughout the observation until its end.
  - The timepoint(s) of interest: End of 6 months from enrollment
  - Name of outcome: User quitting attempts
  - Method of measurement: Number of quitting attempts per patient, including the frequency of the quitting attempts.
  - The timepoint(s) of interest: End of 6 months from enrollment
  - Name of outcome: User app behavior
  - Method of measurement: It will be measured as the time spent per app section.
  - The timepoint(s) of interest: End of 6 months from enrollment
  - Name of outcome: User satisfaction with messages
  - Method of measurement: Patients' answers to a satisfaction questionnaire that will be handed to them at the end of the study.
  - The timepoint(s) of interest: End of 6 months from enrollment
  - Name of outcome: User message ratings
  - Method of measurement: User's votes for each message in a 5 star scale.
  - The timepoint(s) of interest: End of 6 months from enrollment
  - Name of outcome: User lifestyle feedback
  - Method of measurement: Comparison of changes in user lifestyle (at baseline and after 6 months) through the combined questionnaire EQ-5D-5L.
  - The timepoint(s) of interest: End of 6 months from enrollment
  - Name of outcome: User lifestyle feedback
  - Method of measurement: Comparison of changes in user lifestyle (at baseline and after 6 months) through the combined questionnaire IPAQ for physical activity.
  - The timepoint(s) of interest: End of 6 months from enrollment
  - Name of outcome: User lifestyle feedback
  - Method of measurement: Comparison of changes in user lifestyle (at baseline and after 6 months) through the combined questionnaires SF-36.
  - The timepoint(s) of interest: End of 6 months from enrollment
  - Name of outcome: Physical activity
  - Method of measurement: The physical activity would be measured as the total time (minutes) of activity per user, retrieved by GoogleFit in the app.
  - The timepoint(s) of interest: End of 6 months from enrollment
